# Effect of Veneer-Drying Temperature on Selected Properties and Formaldehyde Emission of Birch Plywood

**DOI:** 10.3390/polym12030593

**Published:** 2020-03-05

**Authors:** Pavlo Bekhta, Ján Sedliačik, Nataliya Bekhta

**Affiliations:** 1 Department of Wood-Based Composites, Cellulose, and Paper, Ukrainian National Forestry University, 79057 Lviv, Ukraine; natalijabekhta@gmail.com; 2 Department of Furniture and Wood Products, Technical University in Zvolen, 96001 Zvolen, Slovakia

**Keywords:** birch plywood, veneer-drying temperature, formaldehyde emission, bending strength, modulus of elasticity, bonding strength, thickness swelling, water absorption

## Abstract

In this study, the effect of the veneer-drying process at elevated temperatures on selected properties and formaldehyde emission of plywood panels was determined. We assume that during the veneer drying at high temperatures, more formaldehyde is released from it, and therefore, a lower formaldehyde emission can be expected from the finished plywood. Prior to bonding, birch veneers were dried at 160 °C (control) and 185 °C in an industrial veneer steam dryer (SD) and at 180 °C, 240 °C and 280 °C in an industrial veneer gas dryer (GD). Two types of adhesives were used: urea–formaldehyde (UF) and phenol–formaldehyde (PF) resins. Bonding quality, bending strength and modulus of elasticity in bending, water absorption and thickness swelling of plywood samples were determined. The formaldehyde emission level of samples was also measured. It was concluded from the study that the effects of veneer-drying temperatures on the bonding strength and physical and mechanical properties of plywood panels were significant. Veneer-drying temperatures of 185 °C/SD, 180 °C/GD and 240 °C/GD negatively affected the bending strength and the modulus of elasticity along and across the fibres for both UF and PF plywood samples. Bonding strength mean values obtained from all test panels were above the required value (1.0 MPa) indicated in EN 314-2 standard. The lowest formaldehyde emissions for the UF and PF plywood samples were observed in the samples from veneer dried in a steam dryer at 185 °C/SD.

## 1. Introduction

Plywood and other wood-composite materials such as particle boards, medium- (MDF) and high density fibreboard (HDF) are becoming more popular and are widely used for the manufacture of furniture, cabinets, engineering floors, housing and various construction products. These materials are mainly bonded with thermosetting formaldehyde-type adhesives such as urea–formaldehyde (UF) resin, melamine–urea–formaldehyde (MUF) resin, phenol–formaldehyde (PF) resin, etc. UF adhesives are widely used in the production of wood-based panels because of their excellent adhesion to lignocellulose, excellent internal cohesion, easy processing and application, the lack of colour in the finished product and low cost. However, their poor resistance to external factors, especially moisture, and their tendency to release formaldehyde vapours are significant limitations. Wood-composite materials are the most important sources of formaldehyde emission (FE), a harmful gas when released inside buildings. Because of this, one of the disadvantages of plywood and wood composite materials is their toxicity—the release of formaldehyde. The FE from these materials is indisputable [1]. Adhesives based on UF and PF are carcinogenic in very high concentrations. Therefore, formaldehyde was reclassified in 2004 by the International Agency for Research on Cancer (IARC) as ‘carcinogenic to humans (Group 1)’ [2], compelling companies to reduce FE to lower levels.

Methods for reducing FE in wood-composite materials have been widely discussed over the years, and many of them have been described in [3]. However, a decrease in the formaldehyde content of the resins may impair the bonding strength, and adhesives without formaldehyde can increase the cost of the plywood [4,5].

It is known that natural wood contains and emits volatile organic compounds, including formaldehyde [3,6,7]. Therefore, the issue of FE no longer focuses solely on the adhesive systems used to bond plywood panels. This may also be due to the influence of certain wood species. Typically, coniferous species tend to have a higher content of formaldehyde than hardwood species [7].

It has been reported that heat treatment contributes to the FE from solid wood [8]. Lignin and hemicellulose have the potential for FE, and lignin appears to have more potential in this respect than cellulose and hemicellulose. Moreover, thermohydrolytic processing of wood can lead to FE from polysaccharides [9]. Furthermore, the FE levels depend on numerous factors such as wood species, moisture content (MC), outside temperature, and storing time [9,10,11,12,13,14].

Wood as a natural material contains formaldehyde [6]. The FE from solid wood increases at elevated temperatures and prolonged heating times [9,15], even in the absence of wood resin [16]. In the technological process of manufacturing plywood, the veneer can be subjected to high temperatures in the drying and pressing processes.

Veneer drying is one of the most important stages in the production of wood-based panels, such as plywood and laminated veneer lumber (LVL). As is well known, the purpose of the drying process is to reduce the MC of the veneer to obtain the appropriate values for bonding. Prior to the bonding process, the MC of all veneer sheets should be below 7% [17]. Best bonding results are obtained in plywood panels with veneers having 4–6% MC [11]. The amount of moisture in the wood combined with the water in the adhesives will significantly affect the wetting, spreading, penetration and even curing of the adhesives [18]. Moreover, Aydin et al. [11] reported that the FE from poplar and spruce plywood decreased with increasing veneer MC.

Baldwin [19] stated that the veneer-drying process accounts for some 70% of the thermal energy consumed in plywood production and approximately 60% of the mill’s total energy requirement. The drying temperatures vary mainly from 90 to 160 °C and are normal in the plywood industry. The use of high drying temperatures reduces the drying time of the veneer and increases productivity. The small thickness of veneers allows fast drying. Reducing drying time and energy consumption offers great potential for economic benefits for the woodworking industry [20]. It has been concluded that the practice of high drying temperature could save energy and drying time by 44% and 25%, respectively, compared with normal drying temperature [21]. However, drying to very low MC and at a very high temperature or at a moderate temperature for a long period inactivates the veneer surface, causing poor veneer wetting and therefore poor bonding [22].

In addition, the drying temperature affects both the physical and chemical properties of the veneer surface and therefore the durability and physical–mechanical properties of the plywood decreases [23]. Many studies have been conducted on the effect of veneer-drying temperature on the ability of veneer surfaces to bond [24,25], on the relationship between surface inactivation and bonding strength [22] and optimal conditions for surface preparation [26]. In addition, in recent years, many studies have been conducted on the effect of heat treatment on the properties of plywood panels made from thermally treated veneer [23,27,28,29,30,31,32,33,34].

Some physical and mechanical properties of plywood panels manufactured from alder veneer sheets dried at 20, 110, 150, and 180 °C were determined by Aydin and Colakoglu [23]. These authors concluded that shear strength values of plywood panels decreased and formaldehyde emission increased clearly with increasing veneer drying temperature; no clear difference was found for bending strength of panels manufactured from veneers dried at 110, 150, and 180 °C. Nazerian et al. [27] showed that heat treatment of beech, maple and poplar veneers at 120 or 180 °C for 4 h in a small heating unit under atmospheric pressure alters the physical and chemical properties significantly, but the strength properties begin to deteriorate. In other work [28] poplar veneers were subjected to heat treatment at 80, 130 and 180 °C for 1, 3, and 5 h in a small heating unit under atmospheric pressure. Based on the findings in that study, the bending strength and modulus of elasticity of LVL panels decreased with increasing in temperature and time. Jamalirad et al. [29,30] found that water absorption, thickness swelling, shear and bending strength of plywood samples improve at higher drying temperature 180 °C for 2 h. As we can see, in many of these studies, veneers were subjected to heat treatment at high temperatures for a long time (1–5 h).

In practice, steam and gas dryers are used for veneer drying [35]. In the steam dryer the drying agent is heated by steam and in the gas dryer the drying agent is heated by flue gases from combustion of different types of fuel. Moreover, in the gas dryers (GD), the veneer is dried under severe conditions; the drying temperatures are high (200–280 °C), which significantly shortens the drying time, although there is a risk of extra cracks since the veneer becomes brittle. In the steam dryers (SD), the veneer dries under milder conditions than in gas dryers. Typical drying temperatures are of the order of 160 °C in the steam dryers. There is some information in the literature on veneer drying at temperatures of 100–180 °C, but we did not find any information on veneer drying at high temperatures (180–280 °C), including in gas and steam dryers, and how these high temperatures act for a short period of time on the properties and formaldehyde emission of plywood panels. On the other hand, as mentioned above, heat treatment promotes the release of formaldehyde from solid wood [8].

We hypothesize that during the veneer drying at high temperatures, more formaldehyde is released from it, and therefore, a lower FE can be expected from the finished plywood. Therefore, the purpose of this study was to determine the effects of high temperature drying of veneers on some physical and mechanical properties and formaldehyde emission of plywood panels.

## 2. Materials and Methods

### 2.1. Materials

Birch (*Betula verrucosa* Ehrh.) wood veneers obtained by rotary cutting at industrial conditions were used in the study. Rotary cut veneer sheets with 30 cm by 30 cm dimensions and 1.5 mm thickness were classified into five groups after the peeling process and they were dried at 160 °C (control) and 185 °C in an industrial veneer steam dryer (SD) and at 180 °C, 240 °C and 280 °C in an industrial veneer gas dryer (GD). These drying temperatures were obtained from local producer of rotary-cut veneer and plywood panels. The veneer drying was performed in industrial conditions. Each veneer sheet was dried to 4–6% MC.

Two types of formaldehyde-based adhesives were used in this study: commercial UF and PF resins. UF resin solution used in the manufacturing was composed of 100 parts of UF resin by weight, 15 parts of wheat flour by weight, and 4 parts of hardener by weight. The PF resin was used for plywood panel manufacturing without any filler or additive. The formulations of the adhesive mixtures are given in Table 1.

### 2.2. Plywood Panel Manufacturing

Plywood was processed according to an experimental design, where the factors were the types of adhesive (UF and PF), types of veneer dryer (SD and GD) and temperatures of veneer drying (160 °C, 185 °C, 180 °C, 240 °C and 280 °C) (Table 2). Three-layer and five-layer plywood panels of 300 mm  ×  300 mm were made for the strength and formaldehyde release tests, respectively. Four plywood panels were made for each experimental condition—two panels for the strength test and two panels for the formaldehyde release test. Four control panels were also made for UF and PF adhesives. Totally 48 panels were manufactured.

Plywood panels were made in an electrically heated hydraulic laboratory press. The specific pressing pressure of 1.8 MPa and temperature of 105 °C for UF adhesive and 145 °C for PF adhesive were used, and 5 min (for a three-layer panel) or 6 min (for a five-layer panel) pressing time (during the last 30 s of the press cycle the pressure was continuously reduced to 0 MPa). The glue spread was 160 g·m^−2^ based on wet mass. The adhesive mixture was applied onto one side of every uneven ply. The plies were assembled perpendicularly to each other (veneer sheets were laid up tight/loose) to form plywood of three/five plies. Glue was applied onto the veneer surface with a hand roller spreader.

### 2.3. Panel Testing

During the experiment, all plywood samples were conditioned prior to testing for 2 weeks at 20 ± 2 °C and 65 ± 5% relative humidity. The panels were cut to extract test samples according to the standard requirements. The shear strength was measured according to EN 314-1 [36] and EN 314-2 [37] methods after pretreatment for intended use in interior conditions for UF, and exterior conditions for PF. For the shear strength test, one-half of the samples were tested in dry conditions and the other half in wet conditions. UF plywood test pieces were immersed in water at 20 ± 3 °C for 24 h; PF plywood test pieces were immersed for 4 h in boiling water, then dried in the ventilated oven for 16 h at the temperature of 60 ± 3 °C, then immersed in boiling water for 4 h, followed by cooling in water at 20 ± 3 °C for at least 1 h. Ten samples (a total of 480 samples) were used for each variant both dry and wet shear strength mechanical testing.

Bending strength (MOR) and modulus of elasticity (MOE) tests were carried out for plywood panels manufactured according to EN 310 [38] standard. Twelve samples (a total of 288 samples) were used for the evaluation of plywood MOR and MOE. MOR and MOE in bending were carried out in parallel (‖) and perpendicular (ꓕ) directions, depending on the surface layer.

Dimensional stability in the form of thickness swelling (TS) and water absorption (WA) of the samples were determined according to a water-soaking test based on EN 317 [39], using test pieces of dimension 50 × 50 mm. They were immersed in distilled water for four different periods of 2, 24, 48 and 72 h. After this time the test pieces were removed from the water, weighed, and the thickness was measured. The samples were weighed to the nearest 0.001 g and measured to the nearest 0.01 mm immediately. Six replicate samples were tested for each type of plywood panel. The per cent change from the original thickness represents the TS, and the per cent weight change from the original weight represents the WA.

### 2.4. Formaldehyde Release from Plywood

The formaldehyde release level was measured by means of the desiccator method according to EN ISO 12460-4 [40] standard. The test principle is a determination of the quantity of formaldehyde emitted from plywood samples absorbed in a specified volume of distilled water during 24 h in the glass desiccator.

**Figure 1:**
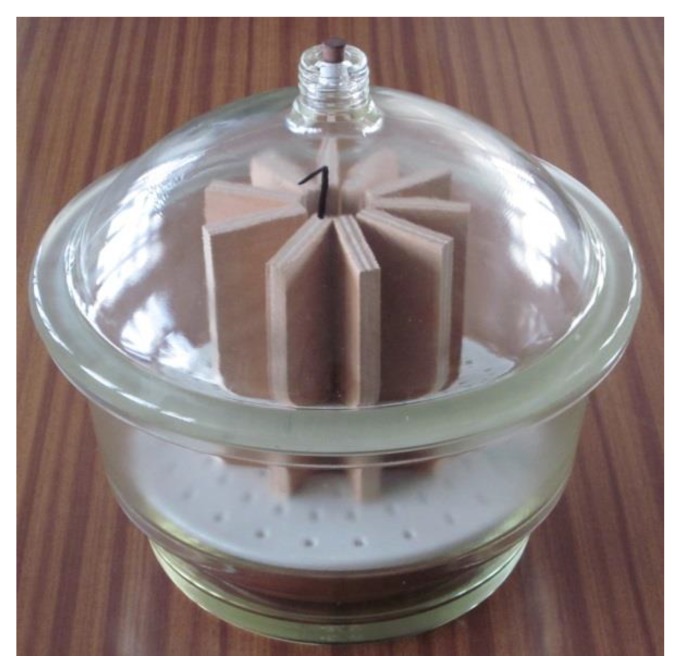
Plywood samples in the glass desiccator.

The test pieces were prepared from each type of plywood with dimensions (150 ± 1) mm × (50 ± 1) mm (length × width) with a total of 1735 cm^2^ (10 pieces). They were then placed in a desiccator with an enclosed volume of 11 litres with a glass crystallizing dish containing 300 mL of distilled water (Figure 1). Samples were removed from the desiccator after 24 h and the obtained formaldehyde solution was prepared for spectroscopic analysis. To determine the formaldehyde content, 25 mL of tested water solution from the desiccator was mixed with 25 mL of acetylacetone-ammonium acetate solution in a 100 mL flask. The stoppered flasks were then heated in a water bath at 65 ± 2 °C for 10 min and subsequently cooled to ambient temperature for 60 ± 5 min. The absorbance of samples was measured on a UviLine SI 5000 spectrophotometer (SI Analytics, College Station, TX, USA) at 412 nm. The formaldehyde content was determined using the calibration curve that was prepared from the standard formaldehyde solutions. The emission tests were carried out in duplicate.

### 2.5. Analysis of Variance

Analysis of variance (ANOVA) at a 0.05 significance level was carried out using IBM SPSS Statistics software (IBM Corp., Armonk, NY, USA) to estimate the relative importance of the effects of the study variables such as types of adhesive and veneer-drying temperature and their interactions on the properties of plywood panels.

## 3. Results and Discussion

### 3.1. Shear Strength of Plywood Samples

Average values of the bonding strength of plywood samples made of birch veneer, dried at high temperatures are given in Table 3. Drying veneer at high temperatures in both steam and gas dryers provides high values of bonding strength of the plywood samples, glued with UF and PF adhesives, compared with control samples. The Duncan test with a 95% confidence level was used to compare the mean values of variance sources and the results for statistical evaluation are presented in Table 3. ANOVA analysis showed that the effect of type of adhesive used, the form of the drying agent and the temperature of the veneer-drying on the bonding strength of plywood samples is statistically significant (*p* ≤ 0.05). Bonding shear strength mean values obtained from the samples of all plywood panels were also above the limit value (1.0 MPa) indicated in EN 314-2 [37] standard. The limit value in the standard indicates the minimum standard requirement for bonding strength. Therefore, the plywood panels tested in this study have met the standard requirement for the bonding strength.

The highest values of bonding strength (2.67 and 2.69 MPa) are observed in plywood samples glued with UF adhesive using veneer dried at temperatures 180 °C/GD and 240 °C/GD. The difference between the bonding strength values for these temperatures is insignificant, and therefore, in practical terms, it is more economically advantageous to use a lower veneer-drying temperature of 180 °C/GD. In contrast, the lowest value of bonding strength (2.48 MPa), not considering the control sample, was recorded for UF plywood samples from veneer, dried at the highest used temperature of 280 °C/GD. For PF plywood samples, the smallest value of bonding strength (1.61 MPa), as in the case of UF plywood samples, was found for plywood samples from veneer dried at the highest applied temperature of 280 °C/GD. This result may be because: (1) excessive veneer drying may cause some drying defects, especially surface cracks [41], which may lead to loss of strength; (2) the thermal processes have caused poor wetting of the wood with adhesive [24,42], and the poor wettability is considered as an indicator of poor bonding strength [43]; (3) at high temperatures during veneer drying extractives migrate to the surface where they concentrate and physically block adhesive contact with wood [22]; (4) the surface roughness increased with increasing veneer-drying temperature; a rough veneer can contribute to the excessive penetration of the adhesive into the wood, increasing the actual (true) surface area while reducing the proportion of the adhesive to that surface [44], as a consequence, there is insufficient adhesive to adhere, resulting in a decrease of bonding quality [45].

Veneer-drying temperatures of 185 °C/SD, 180 °C/GD and 240 °C/GD showed the same effect on the bonding strength; the strength values did not differ significantly. In addition, it can be noted that for UF plywood samples, the bonding strength values for all the investigated temperatures are higher than the value for the control sample. For PF plywood samples, the bonding strength values for the investigated temperatures, except the temperature of 280 °C/GD, are slightly higher than the bonding strength value for the control sample, but the difference between these values is not significant. That is, the veneer-drying temperature of 185 °C/SD, 180 °C/GD and 240 °C/GD did not affect the bonding strength of PF plywood samples. In general, UF plywood samples showed higher values of bonding strength than PF plywood samples. This can be explained by the fact that higher drying temperatures (180–280 °C) initiate degradation of hemicelluloses and formation of acetic acid and formic acid leading to lower pH of veneer surfaces [46,47,48], which can affect the curing of acid-catalysed UF resin and results in improving the shear strength and dimensional stability of plywood samples [29]. Özşahin and Aydin [49] also found that bonding shear strength values of panels manufactured with PF glue were lower than those of panels manufactured with UF glue. They explained this by different pretreatments of plywood samples bonded with UF and PF glues.

Comparing the effect of the veneer-drying temperature on the bonding strength for one type of dryer, it can be noted that the higher temperature 185 °C/SD in the steam dryer had a positive effect on the bonding strength of UF and PF plywood samples compared with the lower temperature of 160 °C/SD in the same dryer. Among the temperatures of 180 °C/GD, 240 °C/GD and 280 °C/GD of gas dryers, the best bonding strength values for UF and PF plywood samples were obtained at the veneer-drying temperatures of 180 °C/GD and 240 °C/GD.

The results obtained by Theander et al. [50] showed that the drying temperatures (180 °C) and degradation of hemicelluloses cause the production of sugar monomers, which play an important role in the bonding quality and in increasing the bonding strength. Fengel and Wegener [51] stated that lignin becomes softer above 160 °C. When lignin softens, it enters the micropores of the wood and makes the woody tissue more homogeneous. This significantly reduces the tendency of wood to swell during soaking and increases bonding strength.

Demirkir et al. [33] reported that the bonding strength values of plywood panels with PF resin increased, as the veneer-drying temperature increased. Jamalirad et al. [30] also showed that the mechanical properties of plywood with increasing drying temperature up to 180 °C for 2 h do not negatively affect shear strength and MOR of plywood samples. Opposing results were obtained in [23], which found that shear strength values of plywood panels decreased clearly with increasing veneer-drying temperature.

### 3.2. Formaldehyde Emission of Plywood Samples

**Figure 2:**
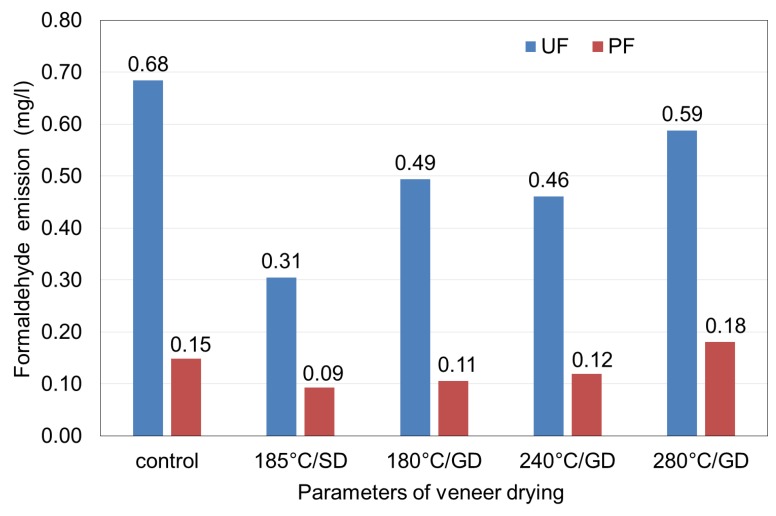
Formaldehyde emission of plywood panels.

FE of plywood samples is expressed as the arithmetic average of the two tests and shown in Figure 2. As can be seen from Figure 2 the values of FE of plywood samples made using veneer dried at high temperatures were lower, except the temperature of 280 °C/GD for PF plywood samples, than the value of FE for control sample. The lowest FE of 0.31 and 0.09 mg/L for the UF and PF plywood samples, respectively, were observed in the samples from veneer dried in a steam dryer at 185 °C/SD. At this temperature, the values of FE are less than 2.2 times and 1.6 times, respectively, for UF and PF plywood samples than those values in the control samples. As can be seen from Figure 2, the veneer-drying temperatures of 180 °C/GD and 240 °C/GD in the gas dryer are more acceptable in terms of reducing FE in plywood samples than the highest veneer-drying temperature of 280 °C/GD. The drying temperature of 280 °C/GD can be considered unacceptable for veneer drying in terms of the bonding strength and FE of plywood samples.

Similar results were obtained by Murata et al. [31] who showed that heating veneer sheets in the temperature range of 150 °C to 170 °C effectively reduced the FE of plywood. Opposing results were obtained by Aydin and Colakoglu [23] who found that FE values of plywood panels increased with increasing veneer-drying temperature.

Hasegawa [8] showed that solid wood dried at high temperatures emits more formaldehyde (HCHO) than that dried at low temperatures. The border of drying temperature, which significantly increases the HCHO emission rate, is dependent on the wood species and its components, e.g., lignin [52,53].

It has been reported in the literature that the amount of organic compounds emitted during thermal drying increases with increasing temperature. The main mechanisms of emission during drying of wood are direct evaporation, steam distillation and thermal degradation. In thermal degradation, high molecular weight organic compounds are cleaved into low molecular weight organic compounds with increasing temperature [51,54].

### 3.3. Bending Strength and Modulus of Elasticity of Plywood Samples

The average values of the MOR and MOE of plywood samples made of birch veneer, dried at high temperatures are shown in Table 4. ANOVA analysis showed that the type of adhesive used, the type of drying agent and the veneer-drying temperature significantly (*p* ≤ 0.05) affect the MOR and MOE of plywood samples.

Mean values obtained for MOR and MOE of plywood panels were higher than the limit values for structural purpose solid wood panels (35 MPa for MOR (‖) and 5 MPa for MOR (ꓕ), 8500 MPa for MOE (‖) and 470 MPa for MOE (ꓕ)) indicated in EN 13,353 [55] for panels having thickness up to 20 mm.

The MOR and MOE values of UF/PF plywood samples from veneer dried at elevated temperatures were (Table 4):

- along the fibres, between 118.1 and 139.7 MPa/131.0–172.8 MPa and 9301.5–10988.3 MPa/9930.4–13924.5 MPa, respectively;
- across the fibres, between 24.2 and 27.8 MPa/25.5–31.2 MPa and 981.9–1351.6 MPa/1197.3–1560.7 MPa, respectively.

Veneer-drying temperatures of 185 °C/SD, 180 °C/GD and 240 °C/GD had a negative effect on the MOR and MOE along and across the fibres for both UF and PF plywood samples. In addition, the values of the MOR and MOE of PF plywood samples are higher than the similar values for UF plywood samples. UF plywood samples made from veneer dried at temperatures of 185 °C/SD and 180 °C/GD have lower values of MOR along and across the fibres than the reference samples. In contrast, UF plywood samples from veneer dried in a gas dryer at temperatures of 240 °C/GD and 280 °C/GD have higher values of MOR along the fibres than control samples. The highest values of MOR along and across the fibres (139.7 and 27.8 MPa, respectively) are observed in UF samples made from veneer dried at the highest drying temperature of 280 °C/GD. PF plywood samples made from veneer dried at temperatures of 185 °C/SD, 180 °C/GD and 240 °C/GD have lower values of MOR along and across the fibres than the reference samples. As in the case of UF samples, the highest value of MOR along and across the fibres are observed in PF samples made from veneer dried at temperature of 280 °C/GD.

High veneer-drying temperatures adversely affected the MOE of plywood samples. The values of the MOE for UF and PF plywood samples, with the exception of MOE (ꓕ) at 280 °C/GD, are lower than the values of the MOE for control samples. In [23] no clear difference was found for MOR of plywood panels manufactured from veneers dried at elevated temperatures. Kol and Seker [34] found that heat treatment had an enhancement effect on MOE and adverse effect on MOR of LVL panels. Nazerian et al. [27] showed that the MOR and MOE of the LVL manufactured from heat-treated poplar veneers decreased with increasing temperature of treatment. Higher weight decrease was obtained from the LVL with veneers treated at 180 °C for 5 h. The optimum drying temperature values were obtained (between 160 and 165 °C) in Scots pine plywood and (between 190 and 196 °C) in alder plywood, for best shear strength, MOR and MOE values [56].

### 3.4. Water Absorption and Thickness Swelling of Plywood Samples

**Figure 3:**
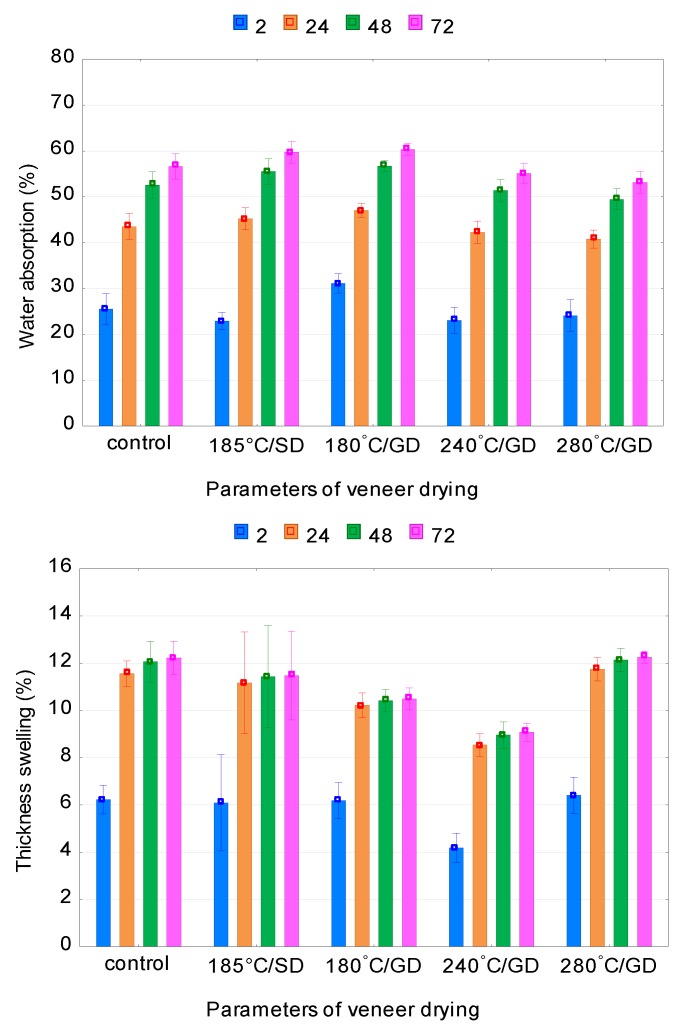
Water absorption and thickness swelling of urea–formaldehyde (UF) plywood samples.

Statistical analysis using ANOVA showed that the type of adhesive, the type of drying agent and the veneer-drying temperature significantly (*p* ≤ 0.05) affected the WA and TS of the plywood samples. The values of TS for the UF plywood samples made from veneer, dried at high temperatures in the steam and gas dryers are less than the TS values of the control samples (Figure 3). The least TS is demonstrated by UF and PF plywood samples from the veneer dried at 240 °C/GD, while the smallest WA is demonstrated by the UF and PF samples from the veneer dried at 240 °C/GD and 280 °C/GD, respectively. With regard to the influence of high temperatures on WA of plywood samples, the picture here is not so straightforward. The WA is greater at drying temperatures of 185 °C/SD and 180 °C/GD, and at drying temperatures of 240 °C/GD and 280 °C/GD the WA is less than the WA of the control samples.

**Figure 4:**
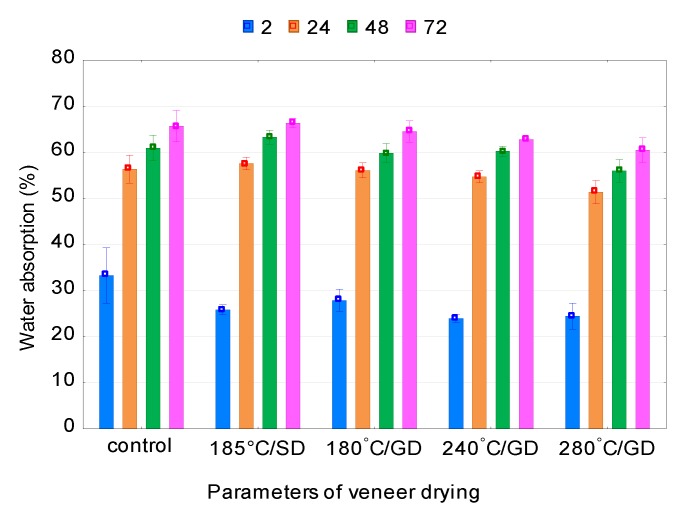

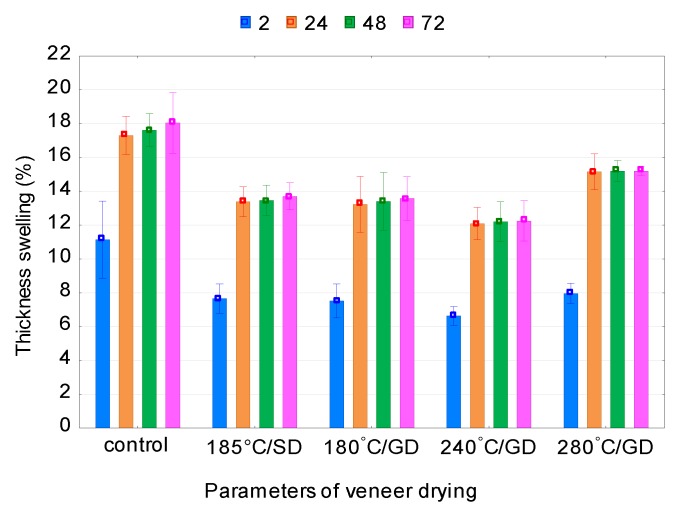
Water absorption and thickness swelling of phenol–formaldehyde (PF) plywood samples.

Drying of veneer at high temperatures reduces the WA and TS of PF plywood samples (Figure 4). The TS values for all PF plywood samples are smaller than for the control samples. The WA values are also smaller for all investigated temperatures, except for a temperature of 185 °C/SD, than for the control samples. The smallest values of WA and TS for PF plywood samples are observed at 280 °C/GD and 240 °C/GD, respectively.

It is known that heat treatment causes a decrease in affinity of the treated material to water. One of the indicators that characterize the affinity of wood to water is the contact angle. Measurements of the contact angle in previous work showed that the contact angle increases on thermally treated veneers, plywood and OSB panels [57,58,59] with the increase in temperature and duration of treatment, indicating a decrease in the affinity of the treated material to water.

Kol and Seker [34] found that heat treatment had an enhancement effect on TS of LVL panel. Nazerian et al. [27] showed that increase of the temperature and treatment time resulted in better dimensional stability for the LVLs manufactured from heat-treated poplar veneers. Jamalirad et al. [29] showed that WA and TS of plywood samples were improved with increasing drying temperature up to 180 °C for 2 h.

It is obvious that with the increase in amount of the heat-treated veneer sheets in a plywood packet the MC decreases. A decrease in MC is expected because the amount of free hydroxyl groups is reduced during heat treatment and the possibility of moisture absorption from the environment is reduced. The equilibrium moisture content (EMC) of the plywood panels decreased with increasing veneer-drying temperature [23]. Several authors [23,46] showed that the EMC values of alder, beech and spruce plywood panels decreased significantly with high-temperature veneer drying.

The improvement in the dimensional stability of the plywood samples from veneer dried at high temperatures is mainly due to the decrease in hygroscopicity due to the chemical changes at high temperatures. Theoretically, existing OH groups in hemicelluloses have the most significant effect on the physical properties of wood. High temperature reduces the EMC of wood [60], the veneer and, as a consequence, TS of plywood panels made from veneer [27]. Zhang et al. [61] showed that changing the character of wood from hydrophilic to more hydrophobic by extracting hemicellulose can also potentially improve the dimensional stability of wood and wood composites.

## 4. Conclusions

In this study, the birch veneer dried at elevated temperatures was successfully used for the bonding of plywood. The results of this study confirmed our assumption that veneer drying at elevated temperatures reduces the FE of plywood. The lowest FE for the UF and PF plywood samples were observed in the samples from veneer dried in a steam dryer at 185 °C/SD. It was also concluded from the study that the effect of veneer-drying temperatures on the bonding strength and physical and mechanical properties of plywood panels was significant. Veneer-drying temperatures of 185 °C/SD, 180 °C/GD and 240 °C/GD had a negative effect on the MOR and MOE along and across the fibres for both UF and PF plywood samples. The highest values of MOR along and across the fibres are observed in UF and PF plywood samples made from veneer dried at the highest drying temperature of 280 °C/GD. Bonding strength means values obtained from all test panels were above the required value (1.0 MPa) according to EN 314-2 standard. The effect of veneer drying at elevated temperatures on the dimensional stability of plywood samples was not as evident as in the case of bonding strength and mechanical properties of samples. The dimensional stability of the PF plywood samples was better than the dimensional stability of the UF samples.

Based on the findings of this study, an optimum veneer-drying temperature of 185 °C/SD could be recommended in industrial application for maintaining a balance between the formaldehyde emissions and bonding strength of the UF- and PF-bonded plywood panels.

## Figures and Tables

**Table 1:** Formulations of adhesive mixtures used for the manufacturing of plywood panels.

	Ingredients of Adhesive	Parts by Weight
Urea–formaldehyde	UF resin (with 66% solid)	100
	Wheat flour	15
	Hardener	4
Phenol–formaldehyde	PF resin (with 47% solid)	100

**Table 2:** Conditions of manufacturing three-layer plywood panels.

Test No.	Manufacturing Conditions						
	Adhesive Type	Type of Drier	Temperature of Veneer Drying (°C)	Adhesive Spread Rate (g/m^2^)	Pressing Pressure (MPa)	Pressing Temperature (°C)	Pressing Time (min)
1	UF	SD	160	160	1.8	105	5/6 *
2		SD	185				
3		GD	180				
4		GD	240				
5		GD	280				
6	PF	SD	160	160	1.8	145	5/6 *
7		SD	185				
8		GD	180				
9		GD	240				
10		GD	280				

* Pressing time for five-layer plywood panels.

**Table 3:** Shear strength of plywood panels.

Drying Temperature (°C)	Moisture Content (%)	Shear Strength (MPa)			
		UF		PF	
		Dry Test	Wet Test	Dry Test	Wet Test
160 °C/SD (control)	6.6	2.27 (0.19) A	1.81 (0.17) A	2.97 (0.24) B	1.78 (0.19) B
185 °C/SD	5.5	3.09 (0.22) C	2.52 (0.12) B	3.23 (0.12) C	1.92 (0.16) B
180 °C/GD	6.2	3.02 (0.28) C	2.67 (0.12) C	2.57 (0.22) A	1.85 (0.10) B
240 °C/GD	4.7	3.08 (0.20) C	2.69 (0.18) C	2.60 (0.21) A	1.93 (0.23) B
280 °C/GD	4.8	2.75 (0.15) B	2.48 (0.21) B	2.56 (0.18) A	1.61 (0.11) A

Values in parentheses are standard deviations. Different letters denote a significant difference. The means followed by the same letter do not statistically differ from each other (*p* ≤ 0.05).

**Table 4:** Duncan’s test results for selected mechanical properties of plywood panels.

Drying Temperature (°C)	Bending Strength (MPa)				Modulus of Elasticity (MPa)			
	UF		PF		UF		PF	
	MOR (‖)	MOR (ꓕ)	MOR (‖)	MOR (ꓕ)	MOE (‖)	MOE (ꓕ)	MOE (‖)	MOE (ꓕ)
160 °C/SD (control)	130.5 (7.1) B	26.2 (2.6) AB	161.0 (9.9) C	31.1 (5.1) C	11,275.5 (905.9) B	1229.6 (92.3) C	14,888.3 (976.9) E	1363.6 (135.6) B
185 °C/SD	118.1 (8.4) A	25.1 (1.8) A	145.8 (11.3) B	29.6 (2.7) BC	9301.5 (898.2) A	1135.3 (64.2) B	11,625.3 (471.3) B	1277.5 (69.6) AB
180 °C/GD	126.3 (4.9) B	24.2 (1.4) A	146.6 (9.9) B	27.0 (3.2) AB	10,852.7 (786.5) B	981.9 (22.0) A	12,686.1 (858.0) C	1232.8 (117.2) A
240 °C/GD	133.6 (10.4) BC	25.5 (2.3) A	131.0 (6.6) A	25.5 (2.3) A	9738.2 (901.5) A	1111.4 (107.6) B	9930.4 (620.3) A	1197.3 (118.1) A
280 °C/GD	139.7 (7.6) C	27.8 (3.0) B	172.8 (11.4) D	31.2 (4.5) C	10,988.3 (743.4) B	1351.6 (99.8) D	13,924.5 (1015.4) D	1560.7 (111.1) C

Values in parentheses are standard deviations. Different letters denote a significant difference. The means followed by the same letter do not statistically differ from each other (*p* ≤ 0.05).
